# The Prevalence, Risk Factors, and Outcomes of Sepsis in Critically Ill Patients in China: A Multicenter Prospective Cohort Study

**DOI:** 10.3389/fmed.2020.593808

**Published:** 2020-12-17

**Authors:** Meiping Wang, Li Jiang, Bo Zhu, Wen Li, Bin Du, Yan Kang, Li Weng, Tiehe Qin, Xiaochun Ma, Duming Zhu, Yushan Wang, Qingyuan Zhan, Meili Duan, Wenxiong Li, Bing Sun, Xiangyuan Cao, Yuhang Ai, Tong Li, Xi Zhu, Jianguo Jia, Jianxin Zhou, Yan He, Xiuming Xi, China Critical Care Sepsis Trial (CCCST) workgroup

**Affiliations:** ^1^Department of Epidemiology and Health Statistics, School of Public Health, Capital Medical University, Beijing, China; ^2^Department of Critical Care Medicine, Fuxing Hospital, Capital Medical University, Beijing, China; ^3^Department of Critical Care Medicine, Xuanwu Hospital, Capital Medical University, Beijing, China; ^4^Medical Intensive Care Unit, Peking Union Medical College Hospital, Beijing, China; ^5^Department of Critical Care Medicine, West China Hospital, Sichuan University, Chengdu, China; ^6^Department of Critical Care Medicine, Guangdong Geriatric Institute, Guangdong General Hospital, Guangdong, China; ^7^Department of Critical Care Medicine, The First Affiliated Hospital of China Medical University, Shenyang, China; ^8^Surgical Intensive Care Unit, Department of Anaesthesiology, ZhongShan Hospital, FuDan University, Shanghai, China; ^9^Intensive Care Unit, The First Hospital of Jilin University, Changchun, China; ^10^Department of Critical Care Medicine, China-Japan Friendship Hospital, Beijing, China; ^11^Department of Critical Care Medicine, Beijing Friendship Hospital, Capital Medical University, Beijing, China; ^12^Surgical Intensive Care Unit, Beijing Chaoyang Hospital, Capital Medical University, Beijing, China; ^13^Department of Respiratory and Critical Care Medicine, Beijing Institute of Respiratory Medicine, Beijing Chaoyang Hospital, Capital Medical University, Beijing, China; ^14^Department of Critical Care Medicine, General Hospital of Ningxia Medical University, Ningxia, China; ^15^Department of Critical Care Medicine, Xiangya Hospital, Central South University, Changsha, China; ^16^Department of Critical Care Medicine, Beijing Tongren Hospital, Capital Medical University, Beijing, China; ^17^Department of Critical Care Medicine, Peking University Third Hospital, Beijing, China; ^18^Surgical Intensive Care Unit, Xuanwu Hospital, Capital Medical University, Beijing, China; ^19^Department of Critical Care Medicine, Beijing Tiantan Hospital, Capital Medical University, Beijing, China

**Keywords:** sepsis, septic shock, mortality, prevalance, risk factor

## Abstract

**Background:** Sepsis is a main cause of morbidity and mortality in critically ill patients. The epidemiology of sepsis in high-income countries is well-known, but information on sepsis in middle- or low-income countries is still deficient, especially in China. The purpose of this study was to explore the prevalence, characteristics, risk factors, treatment, and outcomes of sepsis in critically ill patients in tertiary hospitals in China.

**Methods:** A multicenter prospective observational cohort study was performed with consecutively collected data from adults who stayed in any intensive care unit (ICU) for at least 24 h; data were collected from 1 January 2014 to 31 August 2015, and patients were followed until death or discharge from the hospital.

**Results:** A total of 4,910 patients were enrolled in the study. Of these, 2,086 (42.5%) presented with sepsis or septic shock on admission to the ICU or within the first 48 h after admission to the ICU. ICU mortality was higher in patients with sepsis (13.1%) and septic shock (39.0%) and varied according to geographical region. Acinetobacter, Pseudomonas, and Staphylococcus infections were associated with increased ICU mortality. In addition, age, Acute Physiology, and Chronic Health Evaluation II (APACHE II) scores, pre-existing cardiovascular diseases, malignant tumors, renal replacement therapy (RRT), and septic shock were independent risk factors for mortality in patients with sepsis. The prompt administration of antibiotics (OR 0.65, 95% CI 0.46–0.92) and 30 mL/kg of initial fluid resuscitation during the first 3 h (OR 0.43, 95% CI 0.30–0.63) improved the outcome in patients with septic shock.

**Conclusions:** Sepsis was common and was associated with a high mortality rate in critically ill patients in tertiary hospitals in China. The prompt administration of antibiotics and 30 mL/kg fluid resuscitation decreased the risk of mortality.

## Introduction

Sepsis is a major challenge for public health; it is the main cause of morbidity and mortality in intensive care units (ICUs) and is associated with poor outcomes (1–4). In the United States, the incidence of sepsis is 535 cases per 100,000 person-years and is increasing (5). A meta-analysis of data from high-income countries predicted that 31.5 million sepsis and 19.4 million severe sepsis cases will occur annually worldwide (6). Recently, global data from the Intensive Care Over Nations (ICON) audit showed that 29.5% of patients had sepsis during their ICU stay, and the occurrence rates varied regionally from 13.6 to 39.3% (7).

Although several studies have shown that mortality due to sepsis has declined in the past two decades due to advanced supportive care and the introduction of guidelines, the exact mortality rate is still controversial (8–10). In the United States, mortality due to sepsis decreased from 27.8 to 17.9% between 1979 and 2000 (8). Similarly, in Australia and New Zealand, mortality decreased by almost half (from 35.0 to 18.4%) between 2000 and 2012 (9). However, a multicenter study showed that the in-hospital mortality rate in patients with septic shock was 50.9% in Germany and 58.6% in Italy (10, 11).

To date, there have been few large epidemiological investigations of sepsis in middle- and low-income countries. The ICON audit indicated that low income was associated with increased mortality (12). A multicenter point-prevalence study in Turkey showed that the prevalence of sepsis was 30.8%, with a 75.9% mortality rate in patients with septic shock (13). China is a middle-income country that accounts for one-fifth of the world's population. Information on the epidemiology of sepsis has thus far been limited to particular populations (14–16) or obtained with a cross-sectional study (17), which may not accurately reflect the epidemiology of sepsis in critically ill patients.

The aim of this prospective multicenter observational cohort study, which was conducted by the China Critical Care Sepsis Trial (CCCST) group, was to explore the prevalence, characteristics, risk factors, treatment, and outcomes of sepsis in critically ill patients in tertiary hospitals in China.

## Methods

### Study Design

The CCCST was a prospective multicenter observational cohort study designed to assess the prevalence, characteristics, risk factors, and short- and long-term outcomes in critically ill patients in 18 ICUs in 16 tertiary hospitals in 7 geographical regions from 1 January 2014 to 31 August 2015. Eligible patients who were admitted consecutively to ICUs were aged 18 years or older and stayed in the ICU for at least 24 h. If the patients were admitted to the ICU repeatedly during the same hospitalization event, only the first admission was considered.

### Data Collection

The following variables were extracted from the CCCST dataset: baseline demographic data (age, sex, height, and weight), source and type of admission, main diagnosis, and comorbid conditions. The severity of illness was represented by the Acute Physiology and Chronic Health Evaluation II (APACHE II) score (18) and the Sequential Organ Failure Assessment (SOFA) score (19), which were calculated based on clinical and laboratory values. The APACHE II score was calculated within 24 h of admission to the ICU. The SOFA score was recorded consecutively for 7 days after admission to the ICU, and each component of the SOFA score was evaluated at the onset of sepsis. The use of mechanical ventilation (MV), the use of vasopressors (including dopamine, epinephrine, norepinephrine, and dobutamine), the serum creatinine (SCr) level, and urine output were also continuously recorded for 7 days or until discharge. Other clinical variables, such as blood pressure, arterial partial oxygen pressure (PaO_2_), fraction of inspired oxygen (FiO_2_), nutritional therapy, and renal replacement therapy (RRT), were recorded.

The occurrence, severity, and diagnosis of sepsis were assessed consecutively for 28 days or until discharge. The sites and types of infection, biological samples, and culture results were also recorded. The interval between the first use of targeted antibiotics and the onset of sepsis or septic shock, other antibiotics administered before sepsis and retained pre-sepsis blood cultures were also recorded. The primary outcome was ICU mortality and in-hospital mortality; the lengths of ICU and hospital stays were secondary outcomes.

### Definitions

According to the “Surviving Sepsis Campaign (SSC): International Guidelines for the Management of Sepsis and Septic Shock: 2016” (20), sepsis was defined as life-threatening organ failure (an acute change in the SOFA score ≥2 points) caused by infection on admission to the ICU or within the first 48 h after admission to the ICU. Septic shock was defined as sepsis associated with hypotension that required the administration of vasopressors to maintain a mean arterial pressure >65 mmHg and a serum lactate level >2 mmol/L, despite adequate fluid resuscitation. Community-acquired sepsis was defined as sepsis that was present on admission or that developed within 48 h after hospital admission. Hospital-acquired sepsis was defined as sepsis that developed >48 h after hospital admission. For patients with multiple episodes of sepsis, only the first episode was recorded. Acute kidney injury (AKI) and severity were categorized according to the Kidney Disease: Improving Global Outcomes (KDIGO) guidelines (21). Acute respiratory distress syndrome (ARDS) was defined as a PaO_2_/FIO_2_ ratio <300 mmHg, according to the Berlin definition (22). According to the International Sepsis Forum Consensus Conference on the Definitions of Infection in the Intensive Care Unit, catheter-related sepsis was defined as at least one positive peripheral blood culture with (1) the same microorganism (the same species with the same sensitivities) isolated from the catheter segment or the periphery; (2) the same microorganism isolated from the peripheral blood; or (3) the same organism identified in paired central and peripheral blood cultures, wherein the central blood culture was positive ≥2 h earlier than the peripheral blood culture (23). An unscheduled operation without sufficient preparation within 24 h of the onset of injury was defined as emergency surgery. However, elective surgery was performed after adequate preoperative preparation.

### Statistical Analysis

Continuous variables are presented as the means ± standard deviations (SDs) or medians [interquartile ranges (IQRs)], and categorical variables are presented as percentages. Differences between the groups were compared with the *t*-test, one-way analysis of variance or Wilcoxon rank-sum test for continuous variables and the chi-squared test for categorical variables. Multivariable stepwise logistic regression analysis was used to explore the risk factors for ICU mortality in patients with sepsis or septic shock. Variables with a *p* < 0.2 in the univariate analysis were entered into the multivariable logistic regression analysis. In the multivariable analysis, the centers were included as a random effect. Odds ratios (ORs) with 95% confidence intervals (CIs) were calculated by logistic regression analysis. A two-sided *p* < 0.05 was considered statistically significant. All analyses were performed with IBM SPSS statistical software version 25.0 for Windows (IBM, Armonk, NY, USA) and R version 3.6.1 (http://www.r-project.org).

## Results

### Characteristics of the Participants

**Figure 1:**
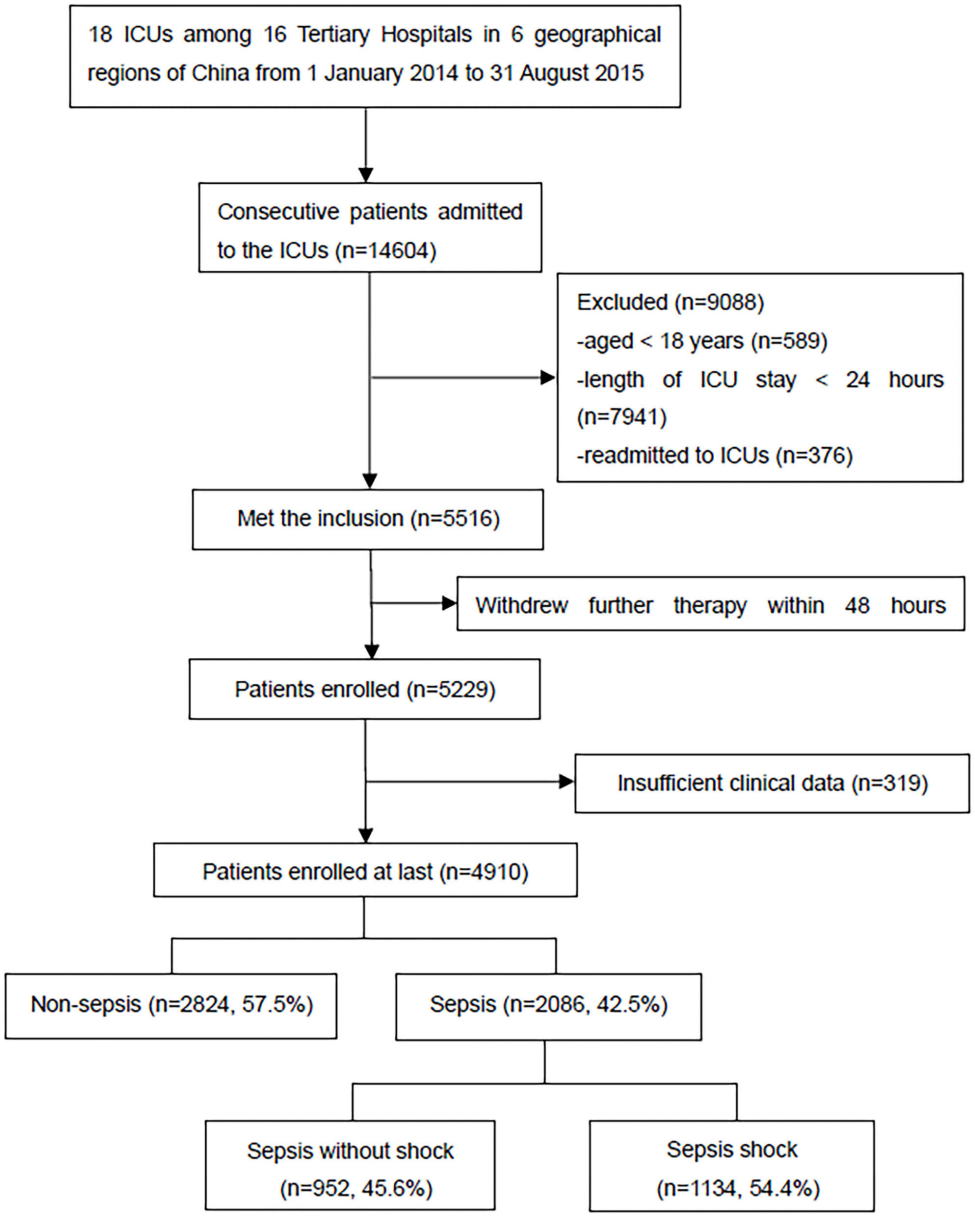
Flowchart of all the participants during the study period. ICU, intensive care unit.

A total of 14,604 critically ill patients were consecutively admitted to the ICUs. A total of 4,910 patients were included in the CCCST. Of those, 2,086 (42.5%) had sepsis, including 1,134 (54.4%) with septic shock (Figure 1). Among all the participants, 3,009 (61.3%) patients had at least one episode of acute organ dysfunction. Patients with sepsis were more likely to be male (65.3 vs. 62.5%), have higher APACHE II (19.0 vs. 13.0) and SOFA (8.0 vs. 6.0) scores, have more comorbid conditions (no comorbidities, 25.1 vs. 38.5%) and organ dysfunction (no organ dysfunction, 22.2 vs. 50.9%), require more MV (77.4 vs. 64.2%) and RRT (18.3 vs. 11.6%), have a longer length of ICU stay (days, 8.0 vs. 4.0) and have higher ICU (27.2 vs. 9.5%) and in-hospital (33.0 vs. 13.1%) mortality rates than those who did not develop sepsis (Table 1). When sepsis was redefined based on the Sepsis-1 definition (see Supplementary Table 1), 200 (4.1%) non-sepsis patients met the definition. The characteristics and outcomes are shown in Supplementary Table 2.

**Table 1:** Baseline characteristics of all the subjects.

	**All patients (*n* = 4,910)**	**Non-sepsis (*n* = 2,824)**	**Sepsis (*n* = 2086)**	***P*-value**
Age, mean ± SD	61.6 ± 17.9	60.9 ± 18.0	62.5 ± 17.8	0.047
Sex, male (%)	3,128 (63.7)	1,766 (62.5)	1,362 (65.3)	<0.001
**Severity on admission**				
APACHE II, median (IQR)	16.0 (10.0–22.0)	13.0 (8.0–19.0)	19.0 (14.0–25.0)	<0.001
SOFA, median (IQR)				
-Initial	8.0 (5.0–11.0)	6.0 (4.0–9.0)	8.0 (5.0–11.0)	<0.001
-Mean	6.0 (4.0–10.0)	5.0 (3.0–8.0)	7.0 (4.0–10.0)	<0.001
-Max	9.0 (6.0–12.0)	7.0 (4.0–10.0)	9.0 (6.0–13.0)	<0.001
Admission diagnosis, *n* (%)				<0.001
Sepsis	1,407 (28.7)	0 (0.0)	1,407 (67.4)	
Pneumonia	859 (17.5)	687 (24.3)	172 (8.2)	
Trauma	295 (6.0)	237 (8.4)	58 (2.8)	
Postoperative monitoring	947 (19.3)	811 (28.7)	136 (6.5)	
Gastrointestinal	374 (7.6)	283 (10.0)	91 (4.4)	
Heart failure	411 (8.4)	322 (11.4)	89 (4.3)	
Neurological	320 (6.5)	266 (9.4)	54 (2.6)	
Others	297 (6.0)	218 (7.7)	79 (3.8)	
**Comorbidities**, ***n*** **(%)**				
Respiratory disease	589 (12.0)	257 (9.1)	332 (15.9)	<0.001
Cardiovascular disease	919 (18.7)	529 (18.7)	390 (18.7)	0.974
Hypertension	1,741 (35.5)	974 (34.5)	767 (36.8)	0.099
Diabetes mellitus	874 (17.8)	463 (16.4)	411 (19.7)	0.003
Chronic renal failure	543 (11.1)	258 (9.1)	285 (13.7)	<0.001
Cancer	499 (10.2)	292 (10.3)	207 (9.9)	0.633
Cirrhosis	119 (2.4)	81 (2.9)	38 (1.8)	0.018
No. of comorbidities, *n* (%)				<0.001
None	1,611 (32.9)	1,087 (38.5)	524 (25.1)	
1	1,932 (39.2)	1,031(36.5)	1,932 (39.2)	
2	834 (17.0)	442 (15.7)	392 (18.8)	
≥3	533 (10.9)	264 (9.3)	269 (12.9)	
**Treatment**				
MV, *n* (%)	3,427 (69.8)	1,841 (64.2)	1,586 (77.4)	<0.001
RRT, *n* (%)	707 (14.4)	332 (11.6)	375 (18.3)	<0.001
Vasopressor, (%)	2,770 (56.4)	1,165 (41.3)	1,605 (76.9)	<0.001
No. of organ failures, *n* (%)				<0.001
0	1,901 (38.7)	1,438 (50.9)	463 (22.2)	
1	1,357 (27.6)	874 (30.9)	483 (23.2)	
2	1,118 (22.8)	465 (16.5)	653 (31.3)	
≥3	534 (10.9)	47 (1.7)	487 (23.3)	
**Length of stay, days, median (IQR)**				
ICU	6.0 (3.0–12.0)	4.0 (2.0–9.0)	8.0 (4.0–16.0)	<0.001
Hospital	17.0 (10.0–27.0)	17.0 (10.0–25.0)	18.0 (10.0–29.0)	0.002
**Mortality**, ***n*** **(%)**				
ICU	834 (17.0)	267 (9.5)	567 (27.2)	<0.001
Hospital	1,058 (21.5)	370 (13.1)	688 (33.0)	<0.001

*SD, standard deviation; APACHE II, Acute Physiology and Chronic Health Evaluation; SOFA, Sequential Organ Failure Assessment; IQR, interquartile range; ICU, intensive care unit; MV, mechanical ventilation; RRT, renal replacement therapy*.

### Distribution of Sites and Isolated Organisms

The most common site of infection in patients with sepsis was the lungs (55.5%), followed by the abdomen (24.4%). Microorganism culture was performed in 1,843 (88.4%) patients, and 1,275 (61.1%) had one or more positive cultures (Table 2). Among the patients with positive isolates, 407 (31.9%) were infected by gram-positive organisms, and 949 (74.4%) were infected by gram-negative organisms. Acinetobacter (29.0%) was the most common gram-negative organism and was usually isolated from the lungs (37.3%), pleura (42.9%), bloodstream (29.7%), catheter-related locations (42.9%), and central nervous system (CNS) (50.0%). Staphylococcus (18.2%) was the most common gram-positive organism and was usually isolated from patients with wound/soft tissue infections (26.8%) (Table 2, Supplementary Table 3). Patients with hospital-acquired sepsis were more likely to have positive cultures of Acinetobacter (34.2 vs. 25.2%) and Pseudomonas (25.1 vs. 19.8). In the patients with septic shock, Pseudomonas (24.1 vs. 19.3%) and Candida (21.8 vs. 19.3%) isolates were likely to be present; Klebsiella isolates were less common than Pseudomonas and Candida (8.9 vs. 15.1%) isolates (Table 2). The microorganism species were significantly different according to region (see Supplementary Table 4).

**Table 2:** Distribution of the most common sites of infection and isolated organism.

	***n***	**Origin of infection**, ***n*** **(%)**			**Severity of sepsis**, ***n*** **(%)**		
		**Community-acquired**	**Hospital-acquired**	***p***	**Sepsis**	**Septic shock**	***P*-value**
Patients with sepsis	2,086	1,181 (56.6)	905 (43.4)		952 (45.6)	1,134 (54.4)	
**Site of infection^#^**							
Lung	1,157 (55.5)	599 (50.7)	588 (61.7)	0.001	521 (54.7)	636 (56.1)	0.534
Pleura	121 (5.8)	87 (7.4)	34 (3.8)	<0.001	75 (7.9)	46 (4.1)	<0.001
Abdomen	510 (24.4)	370 (31.3)	140 (15.5)	<0.001	252 (26.5)	258 (22.8)	0.049
Urinary tract	88 (4.2)	65 (5.5)	23 (2.5)	0.001	49 (5.1)	39 (3.4)	0.053
Bloodstream	157 (7.5)	73 (6.2)	84 (9.3)	0.008	69 (7.2)	88 (7.8)	0.659
Catheter-related sites	42 (2.0)	5 (0.4)	37 (4.1)	<0.001	31 (3.3)	11 (1.0)	<0.001
Wound/soft tissue	107 (5.1)	56 (4.7)	51 (5.6)	0.359	79 (8.3)	28 (2.5)	<0.001
CNS	34 (1.6)	28 (2.4)	6 (0.7)	0.002	21 (2.2)	13 (1.1)	0.057
Unknown	314 (15.1)	152 (12.9)	162 (17.9)	0.001	115 (12.1)	199 (19.5)	0.001
Results of culture				0.007			<0.001
Undetermined	243 (11.6)	153 (13.0)	90 (9.9)		179 (18.8)	64 (5.6)	
Negative	568 (27.2)	294 (24.9)	274 (30.3)		238 (25.0)	330 (29.1)	
Positive	1,275 (61.1)	734 (62.2)	541 (59.8)		535 (56.2)	740 (65.3)	
Isolated organisms^&^	1,275						
Gram-positive	407 (31.9)	243 (33.1)	164 (30.3)	0.391	165 (30.8)	242 (32.7)	0.482
Staphylococcus	232 (18.2)	138 (18.8)	94 (17.4)	0.514	86 (16.1)	146 (19.7)	0.095
Enterococcus	132 (10.4)	85 (11.6)	47 (8.7)	0.094	52 (9.7)	80 (10.8)	0.528
Gram-negative	949 (74.4)	552 (75.2)	397 (73.4)	0.461	412 (77.0)	537 (72.6)	0.073
Acinetobacter	370 (29.0)	185 (25.2)	185 (34.2)	<0.001	156 (29.2)	214 (28.9)	0.926
Escherichia	256 (20.1)	153 (20.8)	103 (19.0)	0.426	105 (19.6)	151 (20.4)	0.732
Klebsiella	147 (11.5)	83 (11.3)	64 (11.8)	0.773	81 (15.1)	66 (8.9)	0.001
Pseudomonas	281 (22.0)	145 (19.8)	136 (25.1)	0.022	103 (19.3)	178 (24.1)	0.041
Fungi^§^	326 (25.6)	189 (25.7)	137 (25.3)	0.863	125 (23.3)	201 (27.2)	0.125
Candida	249 (19.5)	146 (19.9)	103 (19.0)	0.704	88 (16.4)	161 (21.8)	0.018
Aspergillus	25 (2.7)	18 (2.5)	17 (3.1)	0.456	13 (2.4)	22 (3.0)	0.558

# *percentage is not equal to 100 because patients may have one or more sites of infection*;

& *patients may have had more than one organism isolated; CNS, central nervous system*;

§ *Candida, yeasts, Aspergillus, and Pneumocystis carinii were included*.

### Organ Dysfunction and Elements of Sepsis

Among all patients with sepsis, approximately three-fourths (74.4%) had been given antibiotics before the onset of sepsis, and 63.2% retained bacteria in their blood culture. Patients with hospital-acquired sepsis were more likely to have higher sub-SOFA scores in any organ (except the kidneys and CNS) and more likely to have AKI (45.1 vs. 39.1%) but less likely to have ARDS (37.3 vs. 49.4%) than those with community-acquired sepsis. The patients with hospital-acquired sepsis commonly received more antibiotics pre-ICU (79.8 vs. 70.4%), antibiotics within 1 h after the recognition of sepsis (76.5 vs. 53.6%) and enteral nutritional therapy (61.8 vs. 52.3%) (Table 3).

**Table 3:** Organ dysfunction scores, use of antibiotics, and use of nutritional therapy in patients with sepsis.

	**All (*n =* 2,086)**	**Community-acquired (*n =* 1,181)**	**Hospital-acquired (*n =* 905)**	***P*-value**
**SOFA sub-scores, median (IQR)**				
SOFA-respiratory	3 (2–3)	3 (2–3)	3 (2–4)	<0.001
SOFA-cardiovascular	2 (0–4)	2 (1–4)	2 (2–4)	0.012
SOFA-coagulation	0 (0–2)	0 (0–2)	0 (0–1)	<0.001
SOFA-hepatic	0 (0–2)	0 (0–0)	1 (0–2)	0.048
SOFA-renal	0 (0–2)	0 (0–2)	0 (0–2)	0.949
SOFA-CNS	1 (0–3)	1 (0–3)	1 (0–2)	0.805
**Organ dysfunction**, ***n*** **(%)**				
ARDS	924 (44.3)	583 (49.4)	341 (37.7)	<0.001
AKI	870 (41.7)	462 (39.1)	408 (45.1)	0.006
Blood culture pre-antibiotics, *n* (%)	1,318 (63.2)	718 (60.8)	600 (66.3)	0.004
Administration of antibiotics pre-ICU, *n* (%)	1,553 (74.4)	831 (70.4)	722 (79.8)	<0.001
Administration of antibiotics within 1 h of sepsis or septic shock onset, *n* (%)	1,328 (63.7)	636 (53.6)	692 (76.5)	<0.001
Nutritional therapy, *n* (%)				0.006
None	225 (10.8)	147 (12.4)	78 (8.6)	
Pre-ICU	257 (12.3)	131 (11.1)	126 (13.9)	
Post-ICU	1,604 (76.9)	903 (76.5)	701 (77.5)	
Nutrition prescription during ICU, *n* (%)				<0.001
Enteral	907 (56.5)	474 (52.3)	433 (61.8)	
Parenteral	488 (30.4)	313 (34.7)	175 (25.0)	
Enteral + Parenteral	209 (13.1)	116 (12.8)	93 (13.3)	

*SOFA, Sequential Organ Failure Assessment; res, respiratory; CNS, central nervous system; IQR, interquartile range; ARDS, acute respiratory distress syndrome; AKI, acute kidney injury; ICU, intensive care unit*.

Among the patients with septic shock, the non-survivors were older, received more MV and RRT, had a longer length of hospital stay, and were more likely to have ARDS and AKI than the survivors (see Supplementary Table 5). During the first 3 h after admission to the ICU or the onset of septic shock, the survivors seemed more likely to fulfill the resuscitation goal of 30 ml/kg fluid bolus than non-survivors (43.6 vs. 32.4%), although there were no significant differences in intravenous fluid administration between the groups (24.2 vs. 21.1 ml/kg). The survivors were also commonly given antibiotics within 1 h (55.9 vs. 49.3%) of onset (Table 4).

**Table 4:** Early management of patients with septic shock.

	**Survivors (*n =* 692)**	**Non-survivors (*n =* 442)**	***P*-value**
**Intravenous fluid administration, ml/kg**			
- 3 h, median (IQR)	24.2 (11.1–30.6)	21.1 (14.1–30.5)	0.741
- 6 h, median (IQR)	33.8 (16.4–46.5)	35.9 (21.7–50.7)	0.004
- 24 h, median (IQR)	56.7 (38.2–81.5)	63.4 (44.1–95.5)	<0.001
**30 ml/kg initial resuscitation**, ***n*** **(%)**			
During first 3 h	302 (43.6)	143 (32.4)	<0.001
During first 6 h	213 (30.8)	126 (28.5)	0.238
**Vasopressor administration**, ***n*** **(%)**			
During first 3 h	360 (52.0)	254 (57.5)	0.073
During first 6 h	394 (56.9)	281 (63.6)	0.026
During first 24 h	457 (66.0)	315 (71.3)	0.066
Antibiotics within 1 h, *n* (%)	387 (55.9)	218 (49.3)	0.030
Blood culture pre-antibiotics,	501 (72.4)	321 (72.6)	0.753
*n* (%)			

*IQR, interquartile range*.

### Outcomes

**Figure 2:**
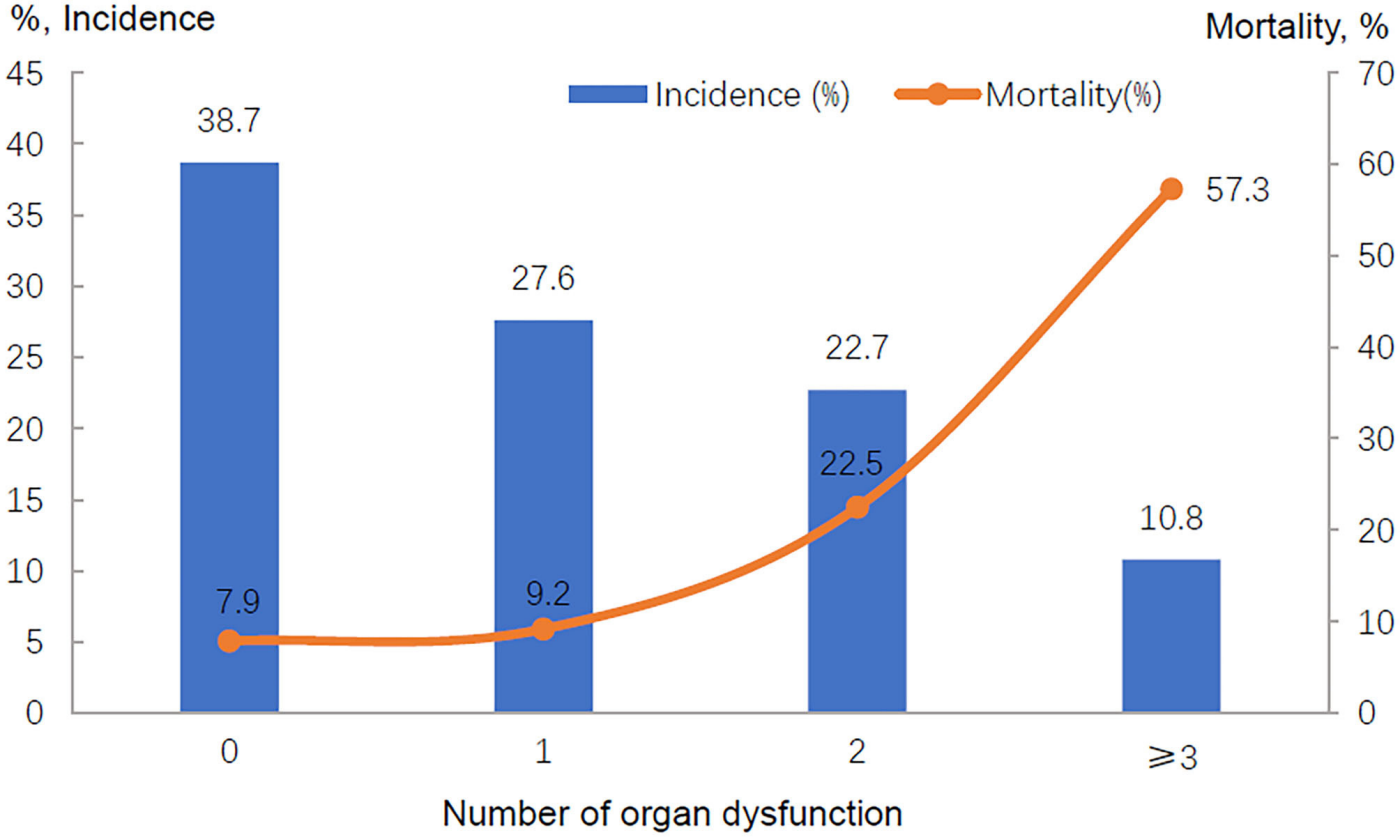
Frequency of organ dysfunction and ICU mortality.

The ICU mortality rates were 27.2% in patients with sepsis and 9.5% in those without sepsis; the in-hospital mortality rates were 33.0 vs. 13.1%, respectively (Table 1). The ICU and in-hospital mortality varied by region (see Supplementary Table 6). In those patients without sepsis, the ICU mortality ranged from 0.8% (Central China) to 13.9% (North China), while in patients with septic shock, the ICU mortality ranged from 20.0% (East China) to 50.0% (Southwest China). There was also a gradual increase in ICU and in-hospital mortality with increasing severity of sepsis (Table 5). Although the patients with septic shock had a decreased length of hospital stay, they did have a significantly increased in-hospital mortality (Table 5). In addition, there was a direct relationship between the number of dysfunctional organs and ICU mortality (Figure 2, Supplementary Figure 1).

**Table 5:** Severity on admission to the ICU, length of stay and mortality in patients with sepsis.

**Category**	***n***	**Severity illness scores**		**Length of stay, days**		**Mortality**, ***n*** **(%)**	
		**APACHE II**	**SOFA**	**ICU**	**Hospital**	**ICU**	**Hospital**
**Onset of sepsis**							
On admission to the ICU	1,407	20.0 (15.0–26.0)	8.0 (5.0–−12.0)	9.0 (4.0–−17.0)	18.0 (10.0–28.0)	32.2 (29.8–34.6)	39.0 (36.5–41.6)
Within 48 h	679	18.0 (13.0–24.0)	8.0 (5.0–11.0)	7.0 (4.0–13.0)	8.0 (10.0–29.0)	16.8 (13.8–19.6)	20.5 (17.4–23.5)
**Severity of sepsis**							
Sepsis without shock	952	17.0 (12.0–22.0)	6.0 (4.0–9.0)	9.0 (4.0–15.0)	19.0 (11.0–29.0)	13.1 (11.0–15.3)	19.3 (16.8–21.8)
Septic shock	1,134	22.0 (16.0–28.0)	9.0 (6.0–13.0)	9.0 (4.0–17.0)	17.0 (9.0–28.0)	39.0 (36.1–41.8)	44.4 (41.6–47.3)
**Original of sepsis**							
Community–acquired sepsis	1,185	19.0 (14.0–25.0)	8.0 (5.0–12.0)	7.0 (4.0 −15.0)	16.0 (9.0–27.0)	26.2 (23.7–28.7)	32.0 (29.3–34.7)
Hospital–acquired sepsis	905	19.0 (14.0–25.0)	8.0 (5.0–11.0)	9.0 (4.0 −17.0)	21.0 (12.0–30.0)	28.5 (25.6–31.5)	34.3 (31.2–37.4)

*APACHE II, Acute Physiology and Chronic Health Evaluation; SOFA, Sequential Organ Failure Assessment; IQR, interquartile range*.

### Factors Associated With Mortality in All Patients With Sepsis

According to the univariate analysis, lung infection was associated with high ICU mortality, and wound/soft tissue infection was associated with low mortality. However, patients with unknown sites of infection did not have elevated mortality rates. Gram-positive (especially Staphylococcus) and gram-negative (Acinetobacter and Pseudomonas) organisms were associated with elevated mortality in patients with positive cultures, whereas Escherichia did not increase ICU mortality (Table 6).

**Table 6:** Associations of sites of infection and isolated organisms with ICU mortality in patients with positive cultures.

**Category**	**Mortality, % (95% CI)**		**Unadjusted ORs (95% CI)**
	**ICU**	**Hospital**	
**Site of infection^#^**			
Lung	34.7 (31.9–37.4)	40.8 (38.0–43.6)	2.44 (1.98–2.99)
Pleura	29.8 (21.5–38.0)	36.4 (27.7–45.1)	1.14 (0.77–1.71)
Abdomen	22.9 (19.3–26.6)	29.6 (25.6–33.6)	0.75 (0.59–1.19)
Urinary tract	28.4 (18.8–38.0)	34.1 (24.0–44.2)	1.07 (0.66–1.71)
Bloodstream	31.8 (24.5–39.2)	36.3 (28.7–43.9)	1.28 (0.90–1.81)
Catheter–related	19.0 (6.7–31.4)	21.4 (8.5–34.4)	0.63 (0.29–1.36)
Wound/soft tissue	11.2 (5.14–17.3)	15.0 (8.1–21.8)	0.32 (0.18–0.60)
CNS	23.5 (8.5–38.6)	26.5 (10.9–42.1)	0.82 (0.37–1.83)
Unknown	14.6 (10.7–18.6)	18.2 (13.9–22.4)	0.41 (0.30–0.57)
**Isolated organisms^&^**			
Gram–positive	45.7 (40.8–50.7)	51.6 (46.6–56.5)	2.18 (1.70–2.78)
Staphylococcus	56.0 (49.6–62.5)	61.6 (55.3–67.9)	3.18 (2.38–4.27)
Enterococcus	40.2 (31.7–48.6)	46.2 (37.6–54.8)	1.37 (0.95–1.99)
Gram–negative	35.1 (32.1–38.1)	41.5 (38.4–44.7)	1.31 (1.00–1.73)
Acinetobacter	44.9 (39.8–50.0)	51.4 (46.2–56.5)	1.99 (1.56–2.65)
Escherichia	24.6 (19.3–29.9)	29.3 (23.7–34.9)	0.59 (0.43–0.80)
Klebsiella	29.9 (22.4–37.4)	36.1 (28.2–43.9)	0.83 (0.57–1.20)
Pseudomonas	49.5 (43.6–55.4)	54.8 (49.0–60.7)	2.39 (1.82–3.13)
Fungi^§^	30.7 (25.6–35.7)	38.3 (33.0–43.7)	0.84 (0.64–1.10)
Candida	33.3 (27.4–39.2)	42.2 (36.0–48.3)	0.99 (0.74–1.32)
Aspergillus	25.7 (10.5–41.0)	28.6 (12.8–44.3)	0.68 (0.21–1.46)

# *percentage is not equal to 100 because patients may have one or more sites of infection*;

& *patients may have had more than one organism isolated; CNS, central nervous system*;

§ *Candida, yeasts, Aspergillus and Pneumocystis carinii were included*.

After adjusting for potential confounders, having no comorbid conditions and the use of antibiotics within 1 h of sepsis onset were associated with relatively lower ICU mortality in patients with sepsis. However, advanced age; high APACHE II score; comorbid cardiovascular diseases and tumors; infection with Acinetobacter, Pseudomonas, and Staphylococcus; and the use of RRT were independent risk factors for ICU mortality in patients with sepsis. Patients with septic shock had a nearly 3-fold increased risk of mortality than those without shock (OR 2.92, 95% CI 2.04–4.17, Table 7). Similarly, the Kaplan-Meier survival curves showed a decreased probability of survival in patients with septic shock than in those without shock (see Supplementary Figure 2). Achieving the resuscitation goal of 30 ml/kg during the first 3 h (OR 0.43, 95% CI, 0.30–0.63) and administering antibiotics within 1 h (OR 0.65, 95% CI, 0.46–0.92) of the onset of septic shock decreased the odds ratio for mortality in patients with septic shock (Table 7).

**Table 7:** Multivariate logistic regression analysis of ICU mortality in patients with sepsis or septic shock.

**Characteristics**	**OR (95% CI)**	***P*-value**
**All patients with sepsis (*****n=*** **2,086)^∮^**		
Age, for per 1 year old	1.04 (1.03–1.05)	<0.001
APACHE II, for per one point increase	1.16 (1.13–1.21)	<0.001
Comorbid condition		
None	0.40 (0.24–0.64)	<0.001
Cardiovascular disease	2.15 (1.45–3.20)	<0.001
Tumor	2.43 (1.39–4.23)	0.002
Septic shock	2.68 (1.84–3.92)	<0.001
Isolated microorganisms		
Acinetobacter	2.21 (1.51–3.25)	<0.001
Pseudomonas	2.09 (1.36–3.23)	0.001
Staphylococcus	2.31 (1.08–3.25)	<0.001
Renal replacement therapy	2.49 (1.72–3.61)	<0.001
Uses of antibiotics within 1 h	0.58 (0.42–0.81)	0.001
**Patients with septic shock (*****n=*** **1,134)^£^**		
Age, for per 1 year old	1.04 (1.03–1.06)	<0.001
APACHE II, for per one point increase	1.17 (1.13–1.20)	<0.001
Isolated microorganisms		
Pseudomonas	2.14 (1.32–3.47)	0.002
Staphylococcus	2.36 (1.96–4.77)	0.001
Renal replacement therapy	3.14 (2.09–4.73)	<0.001
Use of antibiotics within 1 h	0.65 (0.46 −0.92)	0.001
30 mL/kg initial resuscitation during first 3 h	0.43 (0.30–0.63)	<0.001

*APACHE II, Acute Physiology and Chronic Health Evaluation; RRT, renal replacement therapy; CI, confidence interval*;

∮ *adjusted for age; sex; APACHE II score; SOFA-initial score; comorbid conditions; use of MV, RRT and vasopressors; sites of infection; isolated organisms; use of antibiotics within 1 h; use of antibiotics pre-sepsis; and septic shock*.

£ *Adjusted for age; APACHE II score; SOFA-initial score; comorbid cardiovascular diseases, hypertension, diabetes mellitus and chronic renal failure; use of MV, RRT and vasopressors; administration of antibiotics within 1 h; and 30 mL/kg fluid resuscitation within the first 3 h of onset of septic shock or sepsis-induced hypoperfusion*.

## Discussion

This large-scale multicenter prospective cohort study showed a high prevalence of sepsis in Chinese ICUs in tertiary hospitals. More than two-fifths of the patients developed sepsis on admission to the ICU or within 48 h after their ICU admission, more than half was septic shock. And the in-hospital mortality in those patients with septic shock was as high as 44.4%. Furthermore, Acinetobacter and Pseudomonas were the most common isolated microorganisms, and the prompt administration of antibiotics and sufficient fluid resuscitation improved survival.

The prevalence of sepsis in Chinese ICUs was similar to that in another cross-sectional study in China (17) and higher than that in other previous studies (7, 10, 13, 24). Several reasons may explain the differences. First, we excluded those who stayed in the ICU for <24 h. Second, different definitions of sepsis and organ dysfunction might be associated with difference prevalence. Moreover, the type of ICU may affect the prevalence.

In our cohort study, the overall ICU and in-hospital mortality rates were 17.0 and 21.5%, respectively. The patients with sepsis had an elevated mortality rate: more than one-quarter of the patients with sepsis (27.2%) died in the ICU, and one-third of the patients with sepsis (33.0%) died during hospitalization. In patients with septic shock, the mortality rates increased to 39.0% in the ICU and 44.4% in the hospital. These results were similar to those reported by the ICON audit (7), the Sepsis Occurrence in Acutely Ill Patients (SOAP) study (25) and a study in Norway (26) but higher than those reported in other studies (27, 28). These discrepancies may be associated with multiple organ complications, geographical regions and the national income level. Several previous studies have reported that the mortality ranged from 7.0 to 15.0% in sepsis patients with one organ failure, while the mortality reached 45.0–88.6% in patients with four or more organ failures (14, 25, 29). Our study showed that the ICU mortality rate was 9.9% in sepsis patients with one organ failure, while it was 60.8% in those with four or more organ failures. Another finding in our study was that the mortality varied considerable among different geographical regions, which was similar to the result reported by Xie et al. (17). Patients in Southwest China had a relatively high mortality rate, which may be related to the relatively underdeveloped economy. Previous studies have found that a low gross national income was associated with a higher mortality rate (12), especially in low-income countries (30, 31). However, we did not find significantly different outcomes when sepsis was defined using the Sepsis-1 and Sepsis-3 definitions.

As in other studies, the most common site of infection was the lungs in patients with sepsis (15, 17, 25, 32). The frequency of abdominal infection was higher than that reported in previous studies (15, 33, 34). Nevertheless, several national studies reported similar rates of abdominal infection, ranging from 21.3 to 26.0% (24.4% in our study) (17, 25, 35, 36). Hospital-acquired sepsis was more likely to be associated with lung infections and less likely to be associated with abdominal infections than community-acquired sepsis. Recent studies showed that patients with hospital-acquired sepsis had an elevated mortality (37–39). However, we did not find that hospital-acquired sepsis was associated with significantly increased mortality, which may be related to our failure to include ICU-acquired sepsis.

In our study, organisms were isolated from 61.6% of the patients with sepsis; previous epidemiological studies reported both lower (11) and higher (7, 32, 40) rates of positive isolates than that in our study. Over 70% of the patients were infected by gram-negative organisms, while one-third of the patients were infected by gram-positive organisms. Interestingly, Acinetobacter was isolated from 29.0% of the sepsis patients, which was higher than the proportions reported in previous studies (7, 14, 32), possibly because of different control strategies, the use of broad-spectrum antibiotics and the multidrug resistance of Acinetobacter species, especially the high level of resistance to carbapenems. The attention paid to sanitary precautions and the quality of care have improved substantially, but nosocomial infections still occur. In addition, there were significant regional differences in the organisms isolated from the cultures, and the reasons for the variations in the distributions of microorganisms in our study are unknown; these results are probably associated with cultivation techniques and methods.

The SSC 2016 and other studies have recommended the early optimization of antibiotic therapy, and early sufficient fluid resuscitation can improve the outcomes of patients with sepsis or septic shock (20, 41, 42). Our results show that initial antibiotic administration within 1 h of the onset of sepsis decreased ICU mortality by 42% in patients with sepsis (OR 0.58, 95% CI: 0.42–0.81), and early effective fluid resuscitation decreased ICU mortality by more than half in septic shock patients (OR 0.43, 95% CI: 0.30–0.63). However, we were unable to assess the effects of the dose of antimicrobial pharmacokinetics on outcomes in those patients, which is another important factor affecting the outcomes (20, 43, 44). In addition, RRT and pre-existing cardiovascular disease and malignant tumors were also associated with mortality.

This was the first cohort study to investigate the epidemiology of sepsis in critically ill patients in tertiary hospitals in China. The sample size included in the study was very large, which was an obvious strength. Nevertheless, there were still several limitations of this study. First, we excluded patients who stayed in the ICU for <24 h and those who stopped treatment within 48 h of admission to the ICU, which may have resulted in the underestimation of the prevalence and outcomes of sepsis. Second, we recorded only the first episode of sepsis for each patient because the number of patients who had two or more episodes during their ICU stay could not be estimated. Third, some of the data that were collected at the bedside (such as Glasgow coma scale score, urine output and hourly fluid balance) may not have been entered into the medical records. Fourth, the inclusion of two centers with small sample sizes (59 eligible participants) may have affected the results, although we adjusted for center in the statistical analysis. Fifth, the data of feeding strategy were not collected and we could not investigate the association between the feeding approach and outcomes (20). In addition, due to traditional beliefs, some critically ill patients terminated therapy and returned home before improvement or death, which may have led to the underestimation of ICU or in-hospital mortality. However, the proportion of the patients who did so is unknown. Despite several limitations of our research, we report the prevalence, etiology, and prognosis of sepsis and the associated risk factors in critically ill adults in tertiary hospitals in China, providing a basis for further epidemiological and health burden studies of sepsis in China.

In conclusion, sepsis is common in critically ill patients in tertiary hospitals in China and is associated with increased mortality. Acinetobacter, Pseudomonas, and Staphylococcus infections were independent risk factors for ICU mortality. The initial administration of antibiotics within 1 h of sepsis onset and adequate fluid resuscitation decreased the risk of mortality. To reduce the burden of sepsis, national policies are urgently needed. Health-care-associated infection prevention programmes, early detection strategies, and appropriate antibiotic and fluid management strategies for the treatment of sepsis are critical.

## Data Availability Statement

The datasets generated and/or analysed during the current study are available from the corresponding author on reasonable request.

## Ethics Statement

The study protocol was approved by the ethics committees of Fuxing Hospital, Capital Medical University (approval notice number 2013FXHEC-KY018), and all other centers. Written informed consent for participation was not required for this observational survey. The patient records and information were anonymized before analysis.

## Author Contributions

XX conceived of, designed, and supervised the study. MW, WenL, YK, LW, TQ, XM, DZ, YW, QZ, MD, WenxL, BS, XC, YA, TL, XZ, JJ, JZ, and the CCCST workgroup participated in the data collection. MW, LJ, BZ, and YH finalized the analysis, designed the study, and interpreted the findings. MW and LJ wrote the drafts of the manuscript. BZ, BD, and LW interpreted the findings and commented on and helped revise the drafts of the manuscript. All authors read and approved the final manuscript. All authors contributed to the article and approved the submitted version.

## Conflict of Interest

The authors declare that the research was conducted in the absence of any commercial or financial relationships that could be construed as a potential conflict of interest.

## Abbreviations

ICU: intensive care unit
APACHE II: Acute Physiology and Chronic Health Evaluation II
RRT: renal replacement therapy
OR: odds ratio
ICON: Intensive Care Over Nations
SOFA: Sequential Organ Failure Assessment
MV: mechanical ventilation
SCr: serum creatinine
PaO2: arterial partial oxygen pressure
FiO_2_: fraction of inspired oxygen
SSC: Surviving Sepsis Campaign
AKI: acute kidney injury
ARDS: acute respiratory distress syndrome
SD: standard deviation
IQR: interquartile range
CI: confidence interval
SOAP: Sepsis Occurrence in Acutely Ill Patients
ICD: International Classification of Diseases
CRF: case report form
GCS: Glasgow coma scale.
