# Supplementary material for: The Prevalence, Risk Factors, and Outcomes of Sepsis in Critically Ill Patients in China: A Multicenter Prospective Cohort Study
Source: Front Med (Lausanne). 2020 Dec 17;7:593808. doi: 10.3389/fmed.2020.593808 (PMC7774866; doi:10.3389/fmed.2020.593808)
Supplement: Supplementary file 1 [file Data_Sheet_1.docx]

**Additional Tables**

Sepsis-1.0 was defined as having a suspected or confirmed site of infection, ≥ 2 systemic inflammatory response syndrome criteria (SIRS). Severe sepsis was defined as sepsis plus at least one sepsis-induced organ dysfunction. Septic shock was defined as severe sepsis associated with refractory hypotension; despite at least 2 h of adequate volume resuscitation, a systolic blood pressure (SBP) < 90 mmHg or a reduction of ≥40 mmHg from baseline level or a mean arterial pressure < 65 mmHg in the absence of other causes of hypotension or the need for vasopressors to maintain SBP ≥ 90 mmHg.

**Table S1.** The definition of Sepsis-1

| Systemic inflammatory response syndrome criteria (SIRS) ≥ 2 criteria |
| --- |
| Temperature > 38.3°C or < 36°C |
| Heart rate > 90/min |
| WBC count > 12,000/μL or < 4000/μL or Normal WBC count > 10% immature forms |
| Respiratory rate ≧20/min or PaCO_2_ ≦ 32 mmHg, or on mechanical ventilation |
| Severe sepsis: sepsis + at least one organ dysfunction |
| Systolic blood pressure (SBP) < 90 mmHg or mean arterial pressure (MAP) < 65 mmHg or SBP decrease > 40mm Hg from patient’s baseline SBP |
| Creatinine > 2.0mg/dL |
| Acute oliguria (urine output < 0.5 mL/kg/hr for at least 2 hrs) |
| Hyperbilirubinemia (plasma total bilirubin > 2 mg/dL) |
| Thrombocytopenia (platelet count < 100,000/μL) |
| Hyperlactatemia ( > 2 mmol/L or 18.0mg/dL) |
| Coagulopathy (international normalized ratio > 1.5) |
| Acute lung injury with PaO_2_/FIO_2_ (P/F) < 250 mmHg in the absence of pneumonia as infection source |
| Acute lung injury with P/F < 200 in the presence of pneumonia as infection source |
| Septic shock: severe sepsis associated with hypotension |
| SBP < 90 mmHg or MBP < 65 mmHg or SBP decrease > 40mm Hg from baseline |

**Table S2.** Baseline characteristics and outcomes of the patients with sepsis according to sepsis-1

|  | All (n=2286) | Sepsis (n=438) | Severe sepsis (n=619) | Septic shock (n=1229) | P value |
| --- | --- | --- | --- | --- | --- |
| Age, Mean ±SD | 62.3±18.0 | 60.8±18.1 | 62.2±18.3 | 62.9±17.7 | 0.117 |
| Sex, male (%) | 1497 (65.5) | 289 (66.0) | 427 (69.0) | 781(63.5) | 0.066 |
| Severity on admission |  |  |  |  |  |
| APACHEII, median (IQR) | 19.0 (13.0- 25.0) | 15.0 (11.0- 20.0) | 17.0 (12.0- 23.0) | 21.0 (16.0-27.0) | <0.001 |
| SOFA, median (IQR) | 8.0 (5.0- 11.0) | 5.0 (3.0- 8.0) | 8.0 (5.0- 10.0) | 9.0 (6.0- 13.0) | <0.001 |
| Source of admission, n (%) |  |  |  |  | <0.001 |
| Emergency Department | 542 (23.7) | 111 (25.3) | 203 (32.8) | 228 (18.6) |  |
| General wards | 820 (35.9) | 146 (33.3) | 197 (31.8) | 477 (38.8) |  |
| Emergency surgery | 299 (13.1) | 76 (17.4) | 77 (12.4) | 146 (11.9) |  |
| Elective surgery | 202 (8.8) | 54 (12.3) | 84 (13.6) | 64 (5.2) |  |
| Other ICU | 43 (1.9) | 9 (2.1) | 17 (2.7) | 17 (1.4) |  |
| Other hospital | 380 (16.6) | 42 (9.6) | 41 (6.6) | 297 (24.4) |  |
| Comorbidities, n (%) |  |  |  |  |  |
| Respiratory disease | 348 (15.2) | 52 (11.9) | 64 (10.3) | 232 (18.9) | <0.001 |
| Cardiovascular disease | 427 (18.7) | 72 (16.4) | 115 (18.6) | 240 (19.5) | 0.361 |
| Hypertension | 839 (36.7) | 147 (33.6) | 242 (39.1) | 450 (36.6) | 0.184 |
| Diabetes mellitus | 457 (20.0) | 94 (21.5) | 134 (21.6) | 229 (18.6) | 0.215 |
| Chronic renal failure | 299 (13.1) | 41 (9.4) | 57 (9.2) | 201 (16.4) | <0.001 |
| Cancer | 230 (10.1) | 28 (6.4) | 52 (8.4) | 50 (12.2) | 0.001 |
| Cirrhosis | 48 (2.1) | 5 (1.1) | 18 (2.9) | 25 (2.0) | <0.001 |
| None | 640 (28.0) | 162 (37.0) | 214 (34.6) | 264 (21.5) | <0.001 |
| Treatment during ICU |  |  |  |  |  |
| MV, n (%) | 1733 (75.8) | 286 (65.3) | 421 (68.0) | 1026 (83.5) | <0.001 |
| RRT, n (%) | 418 (18.3) | 33 (7.5) | 91 (14.7) | 294 (23.9) | <0.001 |
| Vasopressor, (%) | 2770 (56.4) | 1165 (41.3) | 1605 (76.9) |  | <0.001 |
| Length of stay, days, median (IQR) | |  |  |  |  |
| ICU | 8.0 (4.0 - 16.0) | 5.0 (3.0-11.0) | 7.0 (4.0- 15.0) | 9.0 (4.0- 17.0) | <0.001 |
| Hospital | 18.0 (10.0- 28.0) | 17.0 (10.0- 27.0) | 18.0 (9.0- 28.0) | 19.0 (12.0-29.0) | 0.006 |
| Mortality, n (%) |  |  |  |  |  |
| ICU | 579 (25.3) | 32 (7.3) | 84 (13.6) | 463 (37.7) | <0.001 |
| Hospital | 709 (31.0) | 48 (11.0) | 129 (20.8) | 532 (43.3) | <0.001 |
| SD, standard deviation; APACHE II, Acute Physiology and Chronic Health Evaluation; SOFA, Sequential Organ Failure Assessment; IQR, interquartile range; MV, mechanical ventilation; RRT, renal replacement therapy. ICU, intensive care unit; ARDS, acute respiratory distress syndrome; AKI, acute kidney injure. | | | | | |

**Table S3.** The distribution of isolated organisms in patients with positive cultures according to the type of infection

|  | Lung  (n=786) | Pleura  (n=91) | Abdomen  (n=276) | Urinary tract  (n=68) | Bloodstream  (n=101) | Catheter-related (n=14) | Wound/soft tissue (n=56) | CNS  (n=18) | Unknown  (n=180) |
| --- | --- | --- | --- | --- | --- | --- | --- | --- | --- |
| Gram-positive, n (%) | 262 (33.3) | 34 (37.4) | 105 (38.0) | 27 (39.7) | 46 (45.5) | 7 (50.0) | 32 (57.1) | 4 (22.2) | 34 (37.4) |
| Staphylococcus | 168 (21.4) | 15 (16.5) | 42 (15.2) | 13 (19.1) | 25 (24.8) | 3 (21.4) | 15 (26.8) | 4 (22.2) | 15 (16.5) |
| Enterococcus | 66 (8.4) | 18 (19.8) | 62 (22.5) | 10 (14.7) | 20 (19.8) | 3 (21.4) | 11 (19.6) | 0 (0.0) | 18 (19.8) |
| Gram-negative, n (%) | 602 (76.6) | 78 (85.7) | 217 (78.6) | 52 (76.5) | 78 (77.2) | 8 (57.1) | 41 (73.2) | 15 (83.3) | 78 (85.7) |
| Acinetobacter | 296 (37.7) | 39 (42.9) | 61 (22.1) | 15 (22.1) | 30 (29.7) | 6 (42.9) | 10 (17.9) | 9 (50.0) | 39 (42.9) |
| Escherichia | 122 (15.5) | 15 (16.5) | 94 (34.1) | 26 (38.2) | 26 (25.7) | 2 (14.3) | 10 (17.9) | 1 (5.6) | 15 (16.5) |
| Klebsiella | 104 (13.2) | 16 (17.6) | 37 (13.4) | 7 (10.3) | 15 (14.9) | 1 (7.1) | 6 (10.7) | 2 (11.1) | 16 (17.6) |
| Pseudomonas | 200 (25.4) | 22 (24.2) | 51 (18.5) | 10 (14.7) | 19 (18.8) | 5 (35.7) | 12 (21.4) | 3 (16.7) | 22 (24.2) |
| Fungi^§^, n (%) | 79 (10.1) | 28 (30.8) | 61 (22.1) | 29 (42.6) | 24 (23.8) | 5 (35.7) | 9 (16.1) | 4 (22.2) | 28 (30.8) |
| Candida | 12 (1.5) | 21 (23.1) | 49 (17.8) | 24 (35.3) | 19 (18.8) | 3 (18.8) | 5 (8.9) | 4 (22.2) | 21 (23.2) |
| Aspergillus | 25 (3.2) | 2 (2.2) | 3 (1.1) | 2 (2.9) | 1 (1.1) | 1 (7.1) | 1 (7.1) | 0 (0.0) | 2 (2.2) |

^§^Candida, yeasts, Aspergillus and Pneumocystis carinii were included.

**Table S4.** Types of isolated organisms in patients with positive cultures according to geographical region

| Isolated organisms^$＆^ | Northeast  (n=81) | North China  (n=647) | East China  (n=22) | South China  (n=63) | Central China  (n=24) | Northwest  (n=44) | Southwest  (n=394) | All  (n=1275) | P value |
| --- | --- | --- | --- | --- | --- | --- | --- | --- | --- |
| Gram-positive, n (%) | 27(33.3) | 194 (30.0) | 8 (36.4) | 20 (31.7) | 24 (45.8) | 25 (56.8) | 122 (31.0) | 407 (31.9) | 0.012 |
| Staphylococcus | 12 (14.8) | 135 (20.9) | 2 (9.1) | 12 (19.0) | 2 (8.3) | 10 (22.7) | 59 (15.0) | 232 (18.2) | <0.001 |
| Enterococcus | 12(14.8) | 45 (7.0) | 5 (22.7) | 5 (7.9) | 8 (33.3) | 9 (20.5) | 48 (12.2) | 132 (10.4) | 0.533 |
| Gram-negative, n (%) | 64 (79.0) | 451 (69.7) | 18 (81.8) | 34 (54.0) | 16 (66.7) | 32 (72.7) | 334 (84.8) | 949 (74.4) | <0.001 |
| Acinetobacter | 20 (24.7) | 139 (21.5) | 2 (9.1) | 21 (33.3) | 5 (20.8) | 11(25.0) | 172 (43.7) | 370 (29.0) | 0.013 |
| Escherichia | 20 (24.7) | 117 (18.1) | 11 (50.0) | 2 (3.2) | 7 (29.2) | 5 (11.4) | 94 (23.9) | 256 (20.1) | 0.037 |
| Klebsiella | 13 (16.0) | 69 (10.7) | 5 (22.7) | 1 (1.6) | 2 (8.3) | 8 (18.2) | 49 (12.4) | 147 (11.5) | <0.001 |
| Pseudomonas | 12 (14.8) | 162 (25.0) | 5 (22.7) | 7 (11.1) | 2 (8.3) | 14 (31.8) | 78 (20.1) | 281(22.0) | 0.129 |
| Fungi^§^, n (%) | 18 (22.2) | 165 (25.5) | 4 (18.2) | 20 (31.7) | 3 (12.5) | 10 (22.7) | 106 (26.9) | 326 (25.6) | <0.001 |
| Candida | 17 (21.0) | 123 (19.0) | 3 (13.6) | 16 (25.4) | 3 (12.5) | 4 (9.1) | 83 (21.1) | 249 (19.5) | 0.371 |
| Aspergillus | 1 (1.2) | 24 (3.7) | 0 (0.0) | 2 (3.2) | 0 (0.0) | 2 (4.5) | 6 (1.5) | 35 (2.7) | 0.318 |
| ^$^percentage is not equal to 100 because patients may have one or more sites of infection.^＆^Patients may have more than one organisms isolated. ^§^Candida, yeasts, Aspergillus and Pneumocystis carinii were included. ICU, intensive care unit. | | | | | | | | | |

**Table S5.** Characteristics of the patients with septic shock according to survival status

|  | Survivors  (n=692) | Non-survivors  (n=442) | P value |
| --- | --- | --- | --- |
| Age, Mean ±SD | 59.1±17.6 | 69.5±15.9 | <0.001 |
| Sex, male (%) | 430 (62.1) | 288 (65.2) | 0.303 |
| Severity on admission |  |  |  |
| APACHE II, median (IQR) | 19.0 (14.0- 23.0) | 27.0 (22.0- 31.0) | <0.001 |
| SOFA-initial, median (IQR) | 8.0 (6.0- 11.5) | 11.0 (8.0- 14.0) | <0.001 |
| Comorbidities, n (%) |  |  |  |
| Respiratory disease | 124 (17.9) | 97 (21.9) | 0.095 |
| Cardiovascular disease | 118 (17.1) | 107 (24.2) | 0.003 |
| Hypertension | 217 (31.4) | 196 (44.3) | <0.001 |
| Diabetes mellitus | 108 (15.6) | 104 (23.5) | 0.001 |
| Chronic renal failure | 128 (18.5) | 63 (14.3) | 0.063 |
| Cancer | 80 (11.6) | 61 (13.8) | 0.265 |
| Cirrhosis | 12 (1.7) | 12 (2.7) | 0.263 |
| None | 149 (21.5) | 81 (18.3) | 0.190 |
| Treatment |  |  |  |
| MV, n (%) | 554 (80.1) | 398 (90.0) | <0.001 |
| RRT, n (%) | 98 (14.2) | 173 (39.1) | <0.001 |
| Length of Stay, days (IQR) |  |  |  |
| ICU | 8.5 (5.0-16.0) | 9.0 (4.0- 19.0) | 0.554 |
| Hospital | 14.0 (5.5- 24.0) | 19.0 (10.0- 30.0) | <0.001 |
| ARDS, n (%) | 153 (22.1) | 173 (39.2) | <0.001 |
| AKI, n (%) | 292 (42.2) | 277 (62.7) | <0.001 |

SD, standard deviation; APACHE II, Acute Physiology and Chronic Health Evaluation; SOFA, Sequential Organ Failure Assessment; IQR, interquartile range; MV, mechanical ventilation; RRT, renal replacement therapy. ICU, intensive care unit; ARDS, acute respiratory distress syndrome; AKI, acute kidney injure.

**Table S6.** The mortality of the entire cohort according to geographical region

|  | Northeast | North China | East China | South China | Central China | Northwest | Southwest | P |
| --- | --- | --- | --- | --- | --- | --- | --- | --- |
| All participants | 671 | 2121 | 327 | 449 | 165 | 196 | 981 |  |
| ICU mortality, n (%) | 63 (9.4) | 473 (22.4) | 13 (4.0) | 47 (10.5) | 6 (3.7) | 23 (11.7) | 211 (21.5) | <0.001 |
| Hospital mortality, n (%) | 86 (12.8) | 561 (26.4) | 16(4.9) | 66 (14.7) | 13 (7.9) | 31 (15.8) | 284 (29.0) | <0.001 |
| Non-sepsis | 522 (77.8) | 1069 (50.4) | 266 (81.3) | 241 (53.7) | 126 (76.4) | 125 (63.8) | 475 (48.4) |  |
| ICU mortality, n (%) | 31 (5.9) | 148 (13.9) | 6 (2.3) | 13 (5.4) | 1 (0.8) | 13 (10.4) | 55 (11.6) | <0.001 |
| Hospital mortality, n (%) | 44 (8.4) | 190 (17.8) | 6 (2.3) | 19 (7.9) | 1 (0.8) | 17 (13.6) | 93 (19.5) | <0.001 |
| Sepsis | 76 (11.3) | 329 (15.5) | 46(14.1) | 148 (33.0) | 16 (9.7) | 47 (24.0) | 290 (29.6) |  |
| ICU mortality, n (%) | 10 (13.2) | 50 (15.2) | 4 (8.7) | 12 (8.1) | 0 (0) | 1 (2.1) | 48 (16.6) | <0.001 |
| Hospital mortality, n (%) | 14 (18.4) | 65 (19.8) | 6 (13.0) | 21 (14.2) | 3 (18.8) | 3 (6.4) | 72 (24.8) | <0.001 |
| Septic shock | 73 (10.9) | 723 (34.1) | 15 (4.6) | 60 (13.3) | 23 (13.9) | 24 (12.2) | 216 (22.0) |  |
| ICU mortality, n (%) | 22 (30.1) | 273 (37.8) | 3 (20.0) | 22 (36.7) | 5 (21.7) | 9 (37.5) | 108 (50.0) | <0.001 |
| Hospital mortality, n (%) | 28 (38.4) | 307 (42.5) | 4 (26.7) | 26 (43.3) | 9 (39.1) | 11 (45.8) | 119 (55.1) | <0.001 |
| ICU, intensive care unit. | | | | | | | | |

**Additional Figures**

**Fig S1.** Frequency of organ dysfunction and ICU mortality according to the severity of illness


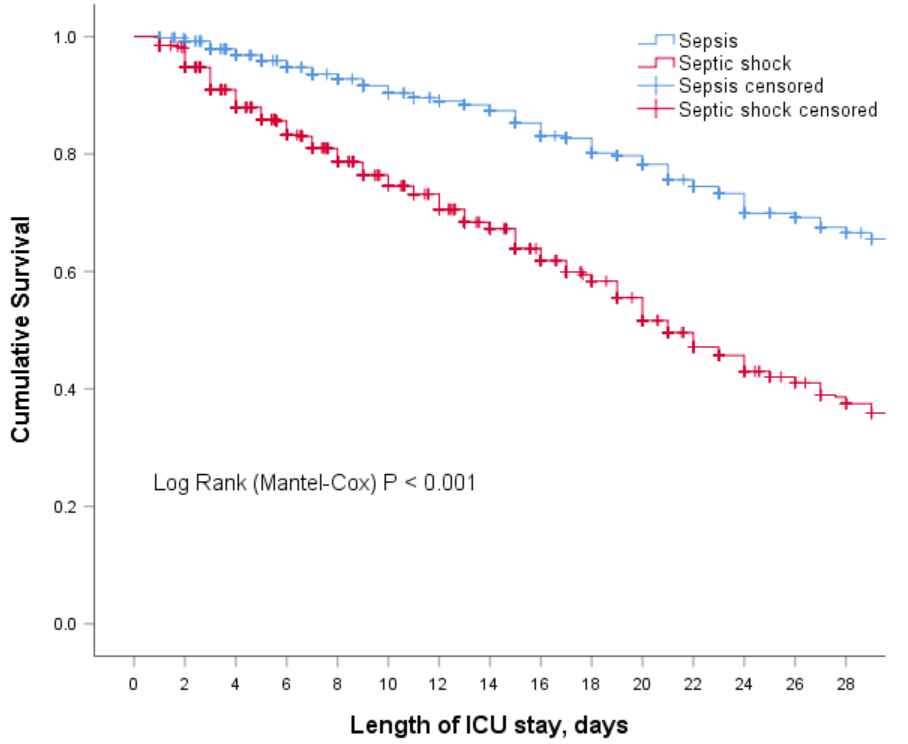


**Fig S2.** Kaplan Meier survival curve of 28-day mortality according to the severity of sepsis
