# Supplementary material for: The Prevalence, Risk Factors, and Outcomes of Sepsis in Critically Ill Patients in China: A Multicenter Prospective Cohort Study
Source: Front Med (Lausanne). 2020 Dec 17;7:593808. doi: 10.3389/fmed.2020.593808 (PMC7774866; doi:10.3389/fmed.2020.593808)
Supplement: Supplementary file 2 [file Data_Sheet_2.docx]

**Additional File 2**

All other ethical bodies that approved our study in the various centers involved:

1. The institutional review board of West China Hospital, Sichuan University;

2. The institutional review board of Peking Union Medical College Hospital;

3. The institutional review board of Guangdong General Hospital;

4. The institutional review board of The First Affiliated Hospital of China Medical University;

5. The institutional review board of ZhongShan Hospital, FuDan University;

6. The institutional review board of The First Hospital of Jilin University;

7. The institutional review board of China-Japan Friendship Hospital;

8. The institutional review board of Beijing Friendship Hospital, Capital Medical University;

9. The institutional review board of Beijing Chaoyang Hospital, Capital Medical University;

10. The institutional review board of General Hospital of Ningxia Medical University;

11. The institutional review board of Xiangya Hospital, Central South University;

12. The institutional review board of Beijing Tongren Hospital, Capital Medical University;

13. The institutional review board of Peking University Third Hospital;

14. The institutional review board of Xuanwu Hospital, Capital Medical University;

15. The institutional review board of Beijing Tiantan Hospital affiliated to Capital Medical University.
