# Supplementary material for: The Prevalence, Risk Factors, and Outcomes of Sepsis in Critically Ill Patients in China: A Multicenter Prospective Cohort Study
Source: Front Med (Lausanne). 2020 Dec 17;7:593808. doi: 10.3389/fmed.2020.593808 (PMC7774866; doi:10.3389/fmed.2020.593808)
Supplement: Supplementary file 3 [file Data_Sheet_3.docx]

**Additional File 3**

Members of the China Critical Care Sepsis Trial (CCCST) workgroup.

1. Department of Critical Care Medicine, Fuxing Hospital, Capital Medical University, Beijing China: Xiuming Xi (xixiuming2937@sina.com), Meiping Wang ([xiaolajiao001326@126.com](mailto:xiaolajiao001326@126.com)), Bo Zhu ([zhubo123@hotmail.com](mailto:zhubo123@hotmail.com)), Qi Jiang (jiangqi7676@sina.com), Zhen Zhao (maggiezhao77@163.com), Hui Song (songhui_321@163.com), Ling Ma (malingbell1982@163.com), Chunyang Li (lcy54@sina.com), Linlin Cao (jitanananhai8510@126.com), Peng Wang ([438867228@qq.com](mailto:438867228@qq.com)); Wen Li (liwen29@sina.com);

2. Li Jiang ([jiangli@sina.com](mailto:jiangli@sina.com)), Department of Critical Care Medicine, Xuanwu Hospital, Capital Medical University, Beijing, China;

3. Ying Wen ([christina7622@163.com](mailto:christina7622@163.com)), Department of Critical Care Medicine, Fuxing Hospital, Capital Medical University, Beitaipingzhuang Community Health Service Centre, Haidian District, Beijing, China;

4. Yibing Zhu (yiyi_bingbing@163.com), Department of Statistics, Fuwai Hospital, National Center for Cardiovascular Diseases, Chinese Academy of Medical Sciences and Peking Union Medical College, Beijing, China.

5. Yan Kang (kangyan33@163.com), Xuelian Liao (xuelianl-liao@hotmail.com), Department of Critical Care Medicine, West China Hospital, Sichuan University, Sichuan, China;

6. Li Weng (wengli@gmail.com), Medical Intensive Care Unit, Peking Union Medical College Hospital, Beijing, China;

7. Tiehe Qin (dr.qin@qq.com), Shouhong Wang, Department of Critical Care Medicine, Guangdong Geriatric Institute, Guangdong General Hospital, Guangdong, China;

8. Xiaochun Ma (xcma2972@sina.com), Liang Wang, Xin Li, Department of Critical Care Medicine, The First Affiliated Hospital of China Medical University, Shenyang, China;

9. Duming Zhu (duming_zhu@163.com), Surgical Intensive Care Unit, Department of Anesthesiology, ZhongShan Hospital, FuDan University, Shanghai, China.

10. Yushan Wang (wang_yushan2010@l63.com), Intensive Care Unit, The First Hospital of Jilin University, Changchun, China;

11. Qingyuan Zhan (Zhanqy0915@yahoo.com.cn), Department of Critical Care Medicine, China-Japan Friendship Hospital, Beijing, China;

12. Meili Duan (13001058598@163.com), Department of Critical Care Medicine, Beijing Friendship Hospital, Capital Medical University, Beijing, China;

13. Wenxiong Li ([lwx7115@sina.com](mailto:lwx7115@sina.com)), Surgical Intensive Care Unit, Beijing Chaoyang Hospital, Capital Medical University, Beijing, China;

14. Bing Sun (ricusunbing＠126. com), Department of Respiratory and Critical Care Medicine, Beijing Institute of Respiratory Medicine, Beijing Chaoyang Hospital, Capital Medical University, Beijing, China;

15. Xiangyuan Cao, Department of Critical Care Medicine, General Hospital of Ningxia Medical University, Ningxia, China;

16. Yuhang Ai (ayhicu1978@sina.com), Department of Critical Care Medicine, Xiangya Hospital, Central South University, Changsha, China;

17. Tong Li (litong@trhos.com), Wei He, Department of Critical Care Medicine, Beijing Tongren Hospital, Capital Medical University, Beijing, China;

18. Yuan Xu (xyuan76@sohu.com), Department of Critical Care Medicine, Beijing Tsinghua Changgung Hospital, Beijing, China;

19. Xi Zhu (xizhuccm@163.com), Department of Critical Care Medicine, Peking University Third Hospital, Beijing, China;

20. Jianguo Jia (jiajianguo_1@126.com), Surgical Intensive Care Unit, Xuanwu Hospital, Capital Medical University, Beijing, China;

21. Jianxin Zhou (jianxinz@yeah.net), Department of Critical Care Medicine, Beijing Tiantan Hospital, Capital Medical University, Beijing, China;

22. Xiaoxia Peng (pengxiaoxia@bch.tom.cn), Centre for Clinical Epidemiology and Evidence-based medicine, Beijing Children's Hospital, Capital Medical University, National Centre for Children Health, Beijing, China.
